# Sub-dimensions of Alcohol Use Disorder in Alcohol Preferring and Non-preferring Rats, a Comparative Study

**DOI:** 10.3389/fnbeh.2019.00003

**Published:** 2019-01-30

**Authors:** Ana Domi, Serena Stopponi, Esi Domi, Roberto Ciccocioppo, Nazzareno Cannella

**Affiliations:** Pharmacology Unit, School of Pharmacy, University of Camerino, Camerino, Italy

**Keywords:** alcoholism, ethanol, self-administration, alcohol-seeking, punished responding, DSM-5

## Abstract

Recent animal models of alcohol use disorder (AUD) are centered in capturing individual vulnerability differences in disease progression. Here, we used genetically selected Marchigian Sardinian alcohol-preferring (msP) and Wistars rats to apply a multidimensional model of AUD adapted from a previously described DSM-IV/DSM-5 multisymptomatic cocaine addiction model. As proof of concept, we hypothesized that msP rats, genetically selected for excessive drinking, would be more prone to develop dependence-like behavior compared to Wistars. Before exposure of animals to alcohol, we monitored basal anxiety in the elevated plus maze (EPM). Animals were then trained in prolonged operant alcohol self-administration, consisting of 30-min daily sessions for 60 days in total. Each session consisted of two 10-min periods of alcohol reinforcement separated by 10-min interval of non-reinforcement. Following training, we applied three criteria of individual vulnerability for AUD: (1) persistence of lever pressing for alcohol when it was not available; (2) motivation for alcohol in a progressive ratio (PR) schedule of reinforcement; and (3) resistance to punishment when alcohol delivery was anticipated by a foot-shock (0.3 mA). We obtained four groups corresponding to the number of criteria met (0–3 crit). Rats in the 0crit and 1crit groups were characterized as resilient, whereas rats in the 2crit and 3crit groups were characterized as prone to develop a dependent-like phenotype. As predicted, the 2–3crit groups were enriched with msP rats while the 0–1crit groups were enriched in Wistar rats. In further analysis, we calculated the global addiction score (GAS) per subject by the sum of the normalized score (z-score) of each criterion. Results showed GAS was highly correlated with animal distribution within the 3 criteria. Specifically, GAS was negative in the 0–1crit groups, and positive in the 2–3crit groups. A positive correlation between basal anxiety and quantity of alcohol intake was detected in msP rats but not Wistars. In conclusion, we demonstrated that the 0/3criteria model is a suitable approach to study individual differences in AUD and that msP rats, selected for excessive-alcohol drinking, show a higher propensity to develop AUD compared to non-preferring Wistars.

## Introduction

Alcohol Use Disorder (AUD) is associated with increased health risks and social harm with dramatic impact to the global disease burden (Rehm, 2011). In 2014, the World Health Organization reported that alcohol contributes to more than 200 diseases, such as alcohol dependence, liver cirrhosis and cancers, as well as alcohol related injuries (WHO, 2014). In attempting to capture the clinical condition of AUDs, a range of procedures have been developed to model alcohol dependence-related traits in rodents (Spanagel, 2000; Tabakoff and Hoffman, 2000; Hopf and Lesscher, 2014). In humans, addictive behavior is characterized by a shift from recreational to compulsive drug seeking as described in the DSM-IV (American Psychiatric Association, 2000). Long-term alcohol consumption induces neuroadaptations that are associated with loss of control, compulsive drug taking and negative emotional states (i.e., anxiety, depression; Wolffgramm and Heyne, 1995; Koob and Le Moal, 1997; Koob, 2013). Particularly, compulsivity, defined by DSM-IV/5 as use of alcohol despite harmful social, health and economic consequences, is a major component in the transition to alcoholism (Spanagel, 2009; Koob and Volkow, 2010; Hasin et al., 2013; McKim et al., 2016). Over the years, the characterization of different lines of rats and mice genetically predisposed to alcohol drinking have helped to elucidate several aspects of AUD neurobiology (McBride and Li, 1998; Bell et al., 2006; McBride et al., 2014). However, a clear understanding of the factors leading the development of dependence itself is still lacking, including compulsivity associated with disease progression (Crabbe, 2010). Such gaps between animal models and the human condition in AUDs are reflected in the limited efficacy of available pharmacological treatments to attenuate compulsive drinking (Volpicelli et al., 1995; Kranzler, 2000; Franck and Jayaram-Lindström, 2013).

Recent research has been oriented towards the development of preclinical models that more closely mimic the complexity of human alcohol addictive behaviors by going beyond the simple alcohol drinking procedures useful to study alcohol reward. By capturing multiple aspects that define AUD such as compulsive alcohol seeking and the inability to abstain from its use despite negative consequences, these models provide new insights into individual vulnerability to develop addictive-like behaviors (Hopf et al., 2011; Radwanska and Kaczmarek, 2012; Seif et al., 2013; Radke et al., 2017; Augier et al., 2018; Giuliano et al., 2018).

One of the features of drug addiction is the inter-individual vulnerability to lose control of drug consumption. This loss of control depends upon genetics, environment, personality traits, psychiatric comorbidities and the interplay of all these factors (Enoch, 2013; Morrow and Flagel, 2016; Egervari et al., 2018). In both humans and laboratory animals the predisposition to develop addiction-like behavior is present in only a small subpopulation of subjects (Anthony et al., 1994; Piazza and Deroche-Gamonet, 2013). In order to identify the inter-individual differences in vulnerability to shift from controlled to compulsive drug intake that define this subpopulation, Deroche-Gamonet et al. (2004) developed a multidimensional animal model of drug addiction (Belin-Rauscent et al., 2016; Deroche-Gamonet et al., 2004; Belin et al., 2008, 2009, 2011). This model characterized a cocaine addiction-prone phenotype in rats, based on the DSM-IV diagnostic criteria of addiction (American Psychiatric Association, 2000), by measuring three traits: (1) inability to refrain from drug seeking; (2) high motivation for the drug; and (3) maintenance of drug use despite negative consequences (Deroche-Gamonet et al., 2004; Kasanetz et al., 2010; Belin et al., 2011). Here, we adapted the DSM-IV/5 based three-criteria model of cocaine addiction to characterize an alcohol-addiction prone phenotype in the rat. We used Marchigian Sardinian alcohol preferring (msP) rats and non-preferring Wistar rats to assess whether a genetic predisposition to ethanol preference contributes to the development of dependence-like behavior. msP rats represent an animal model of genetic predisposition to high ethanol drinking and relapse associated with anxious and depressive-like traits (Ciccocioppo et al., 1999, 2006; Hansson et al., 2006; Cippitelli et al., 2015; Stopponi et al., 2018). Based on these conceptualizations we predicted that msP rats, genetically selected for excessive drinking, would be more prone to develop dependence-like behavior compared to Wistars.

## Materials and Methods

### Animals

Experiments were performed using male Wistar (*n* = 31; Charles River, Calco, Italy) and msP (*n* = 32; bred at the School of Pharmacy, University of Camerino) rats. Rats weighed 200–250 g at the beginning of the study. Rats were housed in pairs under a reversed 12:12-h light/dark cycle (lights off at 9:00 AM) with constant temperature (20–22°C) and humidity (45–55%). Food and water were provided *ad libitum*. Ethanol (95%, Carsetti, Camerino, Italy) was diluted to 10% (v/v) in tap water for chronic, intermittent EtOH exposure and for self-administration behavioral testing. Animals were treated in accordance with the guidelines of the European Community Council Directive for Care and Use of Laboratory Animals. The experimental procedures were approved from the Italian Ministry of Health (authorization n° 414/2016-PR).

### Elevated Plus-Maze

Before being exposed to alcohol, rats were tested in the elevated plus-maze (EPM) to measure anxiety-like traits. The apparatus was constructed of wood and painted black. It consisted of two open arms and two enclosed arms (40 cm high walls) arranged so that the similar arms were opposite each other. The maze, elevated 50 cm above the floor, was located in a sound attenuated room illuminated by a red dim light (~30 lux). The 5 min test began placing the animal in the center of the maze, facing a closed arm. The number of open and closed-arm entries and the time spent in each arm was recorded. Data were expressed in percentage (open or closed time/total time × 100; open or closed entries/total entries × 100). The percentage of time spent in open arms and the number of open arm entries (with entries defined as placement of all four paws into the respective area) were used as measures of anxiety-like behavior, while the number of total arm entries was used as an indicator of general motor activity (Pellow et al., 1985; Cippitelli et al., 2011; Cannella et al., 2016; Domi et al., 2016; Stopponi et al., 2018).

### Alcohol Training Procedure

Prior to operant responding training, rats were exposed to an intermittent two-bottle choice alcohol drinking procedure (choice between 10% alcohol and water) for 3 weeks. This training protocol was adopted to avoid sucrose fading or water deprivation procedures and facilitate the acquisition of operant responding. Alcohol self-administration was performed in rat operant conditioning chambers (Med Associate St Albans, VT, USA) enclosed in sound-attenuating, ventilated, environmental cubicles. Each chamber was equipped with two retractable levers located in the front panel of the chamber with two stimulus lights placed above each lever in addition to a house light and a tone generator. The operant chambers were controlled, and data collected with MED-PC^®^ IV windows-compatible software.

Rats were trained to press the active lever for EtOH 10% on a fixed-ratio 1 (FR1) schedule of reinforcement until a stable baseline was reached (one daily sessions for 7 days). Animals were then moved to a FR3 schedule of reinforcement until addiction criteria were tested. Training sessions were 30 min in duration, during which a 10-min reward-available period (drug-period) was followed by a 10-min reward-unavailable period (no-drug-period) which was followed by a second 10-min drug-period. Pressing the right (active) lever during the drug period resulted in the delivery of 0.1 ml of 10% ethanol in a receptacle connected to a syringe pump, which was followed by the activation of a cue light above the lever for 5 s and a 10 s time-out period. During the no-drug period, signaled by activation of the house light, pressing the active lever had no consequences. Responses on the left or “inactive” lever were recorded during the entire session but did not result in any programmed consequences.

### Evaluation of the Three Criteria for AUD Like-Behavior

At completion of the training we applied three main criteria to monitor individual vulnerability for AUD:

1. *Persistence of response*. We first verified the presence of this behavioral trait in our cohort by running a k-mean cluster analysis on the responses to the “active” lever during the no-drug period from day 1 to day 44. The presence of at least one cluster of subjects escalating lever pressing allowed us to consider this response as a measure of persistence in alcohol seeking. Then, for each subject, “persistence in response” was defined by the individual active lever escalation slope. K-mean analysis and computation of slopes are described in detail in the “Statistical Analysis” section.
2. *Motivation* for alcohol was measured in a progressive ratio (PR) schedule of reinforcement (Cippitelli et al., 2007; Karlsson et al., 2012) in which the response requirement (i.e., the number of lever responses or the ratio required to receive one dose of 10% ethanol) was increased as follows: for each of the first four ethanol deliveries the ratio was increased by 1; for the next four deliveries the ratio was increased by 2 and for all of the following deliveries the ratio was increased by 4 (1, 1, 1, 1, 2, 2, 2, 2, 4, 8, 12, 16, 20, 24, 28, 32, 36, 40, 44, 48, 52, 56, 60, 64, 68, 72 etc.; Economidou et al., 2006). Each alcohol delivery was paired with a 5 s illumination of the cue light. Sessions were terminated when 30 min had elapsed since the last reinforced response. The maximal number of responses that a rat produced to obtain one infusion was referred to as the break point.
3. To measure *resistance to punishment*, rats were placed for 10 min (corresponding to the first drug-period of a standard training session) in the SA chamber were the punishment was a foot-shock (0.3 mA, 0.5 s). The intensity of the shock was chosen based on a pilot study in which different cohorts of msP and Wistar rats were exposed to shock intensities of 0.1 mA, 0.3 mA and 0.6 mA. Results showed that rats were not sensitive to 0.1 mA intensity while at 0.6 mA foot shock completely abolished alcohol self-administration in all the animals. Here, in a FR3 schedule, the first active lever press led to the illumination of a new, different stimulus light (green light), signaling the presence of a shock session. The second active lever press produced a foot-shock of 0.3 mA *via* a metal grid connected to a shock generator. The third active lever press produced the delivery of 0.1 ml of 10% ethanol associated with the cue light. If within a minute, animals did not complete an FR3 the green light turned off and the sequence was reinitiated.

PR and punishment sessions were performed on days 45 and 55 respectively.

A rat was considered positive for a particular addiction-like criterion when the score for this behavior was in the top 34% percent of the distribution. This criterion was arbitrarily chosen based on seminal work from Deroche-Gamonet et al. (2004) and considering that a change of the selection threshold from 25 to 40% has minimal effect on individual rat-group allocation (Deroche-Gamonet and Piazza, 2014). We obtained four groups of rats (0crit, 1crit, 2crit and 3crit) defined by the number of positive criteria met.

As a second level of analysis, we measured the global addiction score (GAS) by calculating the sum of the normalized score (z-score) of each criterion for each subject (Belin et al., 2009).

### Statistical Analysis

Data are expressed as mean ± standard error (SEM). All behavioral experiments were analyzed by mean of Student’s *t*-test comparison, one-way, factorial or repeated-measures analysis of variance (ANOVAs) and covariance (ANCOVA) according to experimental design. We examined for significant violations for assumptions of homogeneity of variances by using Levene’s and Bartlett’s test. In case of deviation from homogeneity of the variance was significantly detected, the Mann-Whitney and Kruskal Wallis non-parametric analysis were used (footshock resistance vs. genotype) and (footshock resistance vs. 0, 1, 2 and 3 criteria), respectively. Significant difference was set at *p* < 0.05. *Post hoc* comparisons were carried out by Newman-Keuls test when appropriate. To asses the escalation of alcohol seeking during the no-drug period we used a k-means cluster analysis with 10 iterations and with maximization of distances between groups defined *a priori* as 3. This approach was taken to verify the existence of a subgroup of animals that increased “active” lever presses over time (from day 1–44). Moreover, for each rat we calculated the slope of “active” responses during the no-drug period over the 44 days (divided in four intervals of 11 days each). Positive values of the slope represent an increase in lever presses over time while negative values reflect a decrease in lever presses over time (Dilleen et al., 2012; Ducret et al., 2016).

## Results

### Anxiety-Like Behavior in msP and Wistar Rats

**Figure 1:**
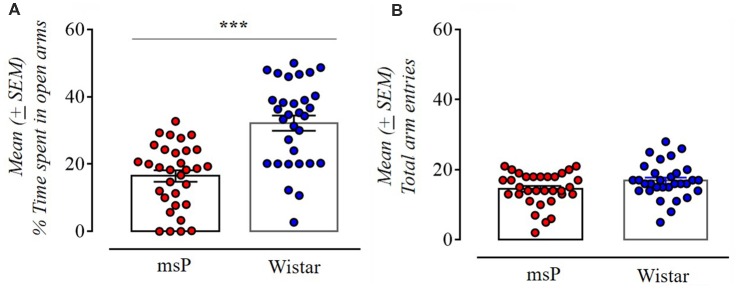
Anxiety-like behavior of Wistar (*n* = 31) and Marchigian Sardinian alcohol preferring (msP) rats (*n* = 32) assessed in the elevated-plus maze (EPM) test. Values are presented as mean percent (%, ±SEM) of open arm time **(A)** and mean (±SEM) number of total arm entries **(B)**. ^***^p < 0.001 between msP and Wistar rats.

The msP rats exhibited significantly higher anxiety-like behavior spending less time in the open arms compared to Wistar rats (*t*_(61)_ = 5.62, *p* < 0.001; Figure 1A). No effect was found in the total number of entries indicating no difference in locomotion (*t*_(61)_ = 1.88, NS; Figure 1B).

### Acquisition of Alcohol Self-Administration in msP and Wistar Rats

**Figure 2:**
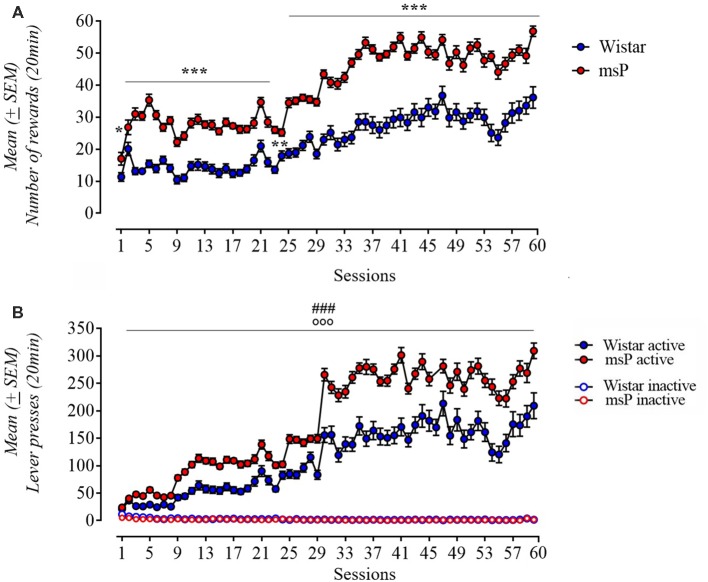
**(A)** Acquisition pattern (60 days) of ethanol 10% (0.1 ml/reward) self-administration in msP (*n* = 32) and Wistar (*n* = 31) rats under a FR-1 (day 1–8) and fixed ratio-3 (FR-3; day 9–60). **(B)** Number of active and inactive lever presses in both strains under a FR-1 (day 1–8) and FR-3 (day 9–60). Values are presented as mean (±SEM). ^***^p < 0.001 significant as compared to msP and Wistar rats in the number of reinforcers (day 3–23 and 24–60), ^**^p < 0.01 (day 24), ^*^p < 0.05 (day 2). ^###^*p* < 0.001, significant as compared to msP and Wistar rats in active lever presses. °°°*p* < 0.001 (active lever vs. inactive lever).

Rats were trained to self-administer alcohol for 60 days. Both msP and Wistar rats acquired and maintained stable alcohol self-administration levels. ANOVA of number of rewards earned revealed a significant effect of line (*F*_(1,61)_ = 83.3; *p* < 0.001), significant effect of session (*F*_(59,3599)_ = 100.31; *p* < 0.001) and a significant line × session interaction (*F*_(53,3599)_ = 5.71; *p* < 0.001). msP rats self-administered significantly more ethanol compared to Wistars. *Post hoc* Neuman-Keuls test (Figure 2A) revealed a significand difference in the following days: day 2 (*p* < 0.05), 24 (*p* < 0.01), 3–23 and 25–60 (*p* < 0.001). Analysis of lever responses over training demonstrated that both msP and Wistar rats discriminated between active and inactive lever and the difference between rat lines was specific to the active lever [line (*F*_(1,60)_ = 39.32; *p* < 0.001), lever (*F*_(1,60)_ = 709.05; *p* < 0.001), session (*F*_(59,3540)_ = 138.15; *p* < 0.001) rat line × lever × session interaction (*F*_(59,3540)_ = 7, 65; *p* < 0.001); Figure 2B].

### Evaluation of the Three Criteria AUD Like-Behavior

#### Persistence in Alcohol Seeking

**Figure 3:**
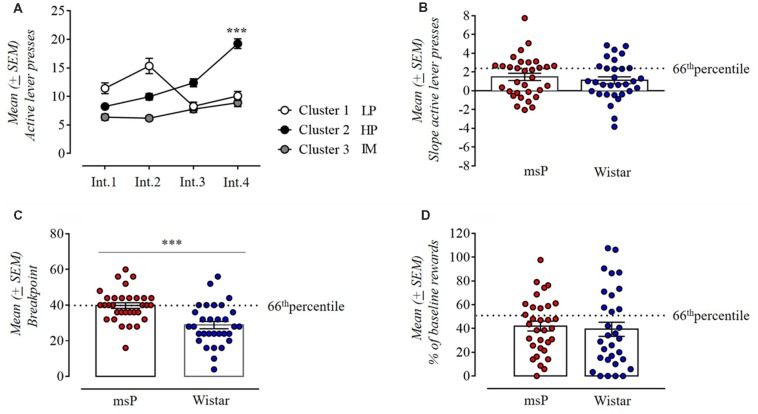
**(A)** K-means clustering with three clusters of the total population (msP and Wistar rats) used to classify animals based on their performance in the drug free period and using as variables the 44 daily sessions divided in four intervals of 11 days each. Cluster 1 defined as low persistent (LP; 14 cases: 8 msP and 6 Wistar rats); cluster 2 defined as high persistent (HP; 20 cases: 12 msP and 8 Wistar rats); cluster 3 defined as intermediate (29 cases: 12 msP and 17 Wistar rats). Values are presented as mean (±SEM). ^***^p < 0.001, significant between Int.1 and Int.4 in Cluster 2. **(B)** Slope values of the number of active lever presses during the drug free period in msP and Wistar rats over the 44 daily sessions divided in four intervals of 11 days each (Int.1, Int.2, Int.3 and Int.4 as points of the slope line). Values are presented as mean (±SEM). **(C)** Motivation for 10% ethanol measured by the break point during a progressive-ratio (PR) schedule of reinforcement in msP and Wistar rats. Values are presented as mean (±SEM). ^***^p < 0.001, significant as compared to msP and Wistar rats in the break point achieved. **(D)** Number of reinforcements in a 10 min foot-shock session for msP and Wistar rats represented as percentage of the baseline (average of the first 10 min of days 42–44). Values are presented as mean (±SEM). The dotted line indicates the 66th percentile of distribution of **(B–D)**.

Persistence in alcohol seeking was measured as the number of active lever presses occurring during the 10 min of non-drug availability. We used a k-means cluster analysis with the number of clusters set *a priori* to three and the variables defined as the 44 days of self-administration (i.e., prior to the PR and foot-shock session tests) divided into 4 intervals of 11 days each (Int.1 = day 1–11, Int.2 = day 12–22, Int.3 = day 23–33, Int.4 = day 34–44; Figure 3A). In assessing whether during the 44 days of operant training animals had progressively increased their lever presses during the drug free period, ANOVA revealed a significant difference between intervals (*F*_(3,180)_ = 22.02; *p* < 0.001) and a significant interval × cluster interaction (*F*_(6,180)_ = 26.06; *p* < 0.001). Subjects in cluster 1 (14 cases: 8 msP and 6 Wistar rats) decreased their lever presses over time and were defined as low persistent (LP). Animals in cluster 2 (20 cases:12 msP and 8 Wistar rats) markedly increased persistence to response over time as revealed by the *post hoc* Neuman-Keuls analysis (Int.1 vs. Int. 4 *p* < 0.001) and were defined as high persistent (HP). Subjects in Cluster 3 (29 cases: 12 msP and 17 Wistar rats) defined as Intermediate (IM) maintained a consistent rate of lever presses during the 44 days of operant training. For the subsequent measure of this criterion, we considered for each rat the slope of lever presses during the no-drug period over the four intervals of time and compared it between msP and Wistars (Figure 3B). Despite the fact that msP rats had higher slope values in average (msP = 1.47 ± 0.39, Wistar = 1.11 ± 0.38), the Student *t*-test revealed no difference between msP and Wistar rats in persistence to response (*t*_(61)_ = 0.67, *p* = ns).

#### Motivation

In PR contingency we compared the breakpoint reached by msP and Wistars by ANCOVA using the average intake during the last three self-administration sessions as covariate. ANCOVA found no significant effect of line (*F*_(1,60)_ = 7.8; *p* = NS). However, the breakpoint was higher in msP rats than Wistar rats (msP = 39.75 ± 1.6, Wistar = 28.84 ± 2.06), indicating a higher motivation to self-administer alcohol in the alcohol preferring line. Indeed, since a higher motivation prompts the msP to self-administer higher amount of alcohol both during the acquisition and the test phase, it is not surprising that using the alcohol intake as covariate would cancel the difference in break point. Confirming this interpretation, when intake is not used as covariate a strong significant difference in break point between the two lines is detected (*t*_(61)_ = 4.2, *p* < 0.001; Figure 3C).

#### Resistance to Punishment

In the punished reward test, animals were presented an aversive stimulus (foot-shock) associated with subsequent administration of alcohol. Observed at group level, punishing of operant responding decreased the motivation for alcohol in both genotypes, but at the individual level the number of rewards self-administered spanned from 0 to 100% of baseline (Figure 3D). The average rate of rewards earned in the punished reward tests were 42% ± 4.15 for msP and 39% ± 3.95 for Wistars calculated as the average of the first 10 min of the last four baseline sessions vs. the first 10 min of the punished schedule session. Mann-Whitney *U*-test revealed no difference between genotypes in alcohol seeking despite punishment expressed in percentage of their baseline (*U* = −0.85, two-sided exact *p* = 0.4).

### Distribution of msP and Wistar Rats by Their Addiction-Like Behavior Score

**Figure 4:**
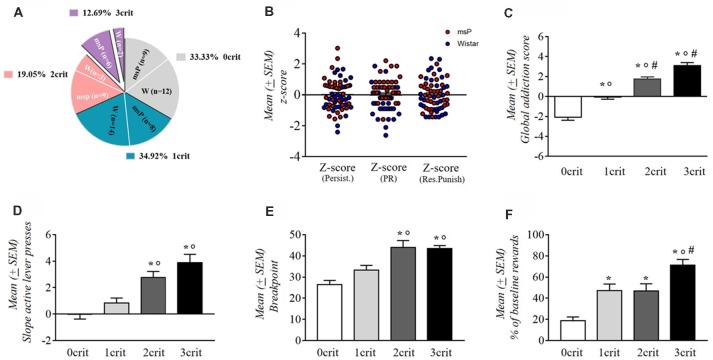
**(A)** Percentage of the total population (*n* = 63) of rats positive for zero (0crit), one (1crit), two (2crit) or three (3crit) addiction like criteria. **(B)** Normalized scores (z-scores) for each of the three criteria in msP and wistar rats. Values are presented as mean (±SEM). **(C)** Addiction score of 0crit, 1crit (addiction resistant rats) 2crit and 3crit (addiction prone rats). Values are presented as mean (±SEM). ^*^p < 0.001, significant as compared to 0crit and 1crit, 2crit, 3crit rats in the global addiction score (GAS). °*p* < 0.001 significant as compared to 1crit and 2crit, 3crit rats in the GAS, ^#^*p* < 0.05 significant as compared to 2crit vs. 3crit rats in the GAS. **(D)** Persistence of response for alcohol during the no-drug period. Values are presented as mean (±SEM). ^*^p < 0.001, significant as compared to 0crit and 2crit, 3crit rats. °*p* < 0.01 significant as compared to 1crit and 2crit rats and °*p* < 0.001 as compared to 1crit and 3crit rats. **(E)** Motivation for alcohol during the PR schedule. Values are presented as mean (±SEM). ^*^p < 0.001, significant as compared to 0crit and 2crit, 3crit rats. °*p* < 0.05 significant as compared to 1crit and 2crit rats and °*p* < 0.01 as compared to 1crit and 3crit rats. **(F)** Resistance to punishment during the punished reward test. Values are presented as mean (±SEM). ^*^p < 0.001 significant as compared to 0crit and 3crit rats. ^*^p < 0.01, significant as compared to 0crit and 1crit, 2crit rats. °*p* < 0.01 significant as compared to 1crit and 3crit rats, ^#^*p* < 0.05 significant as compared to 2crit and 3crit rats.

msP and Wistar rats were scored for each addiction-like behavior and were assigned to a “positive criterion” subgroup if their individual score was in the top of 34% of the total distribution. Rats were then separated into four groups depending on the number of positive criteria met (from 0crit to 3crit). msP rats represented the majority of the 3crit [12.69% of the whole rats’ cohort, 9.52% were msP (*n* = 6) and 3.17% were Wistars (*n* = 2)] and the 2crit groups [19.05% of the whole rats’ cohort, 14.19% were msP (*n* = 9) and 4.76% where Wistars (*n* = 3)]. Conversely, Wistar rats represented the majority of the 1crit [34.92% of the whole rats’ cohort; 12.70% were msP (*n* = 8) and 22.22% were Wistars (*n* = 14)] and the 0crit groups [33.33% of the whole rats cohort; 14.29% were msP (*n* = 9) and 19.05% were Wistars (*n* = 12); Figure 4A]. The criteria for which 1crit and 2crit rats were positive are shown in Table 1.

**Table 1:** Description of the positive criteria met by msP and Wistar rats within the 1crit and 2crit groups.

		**Persistence to response**	**Motivation**	**Resistance to punishment**
1 crit	Wistar	4	3	6
	msP	1	5	2
		**Persistence to response and Motivation**	**Persistence to response and Resistance to punishment**	**Motivation and Resistance to punishment**
2 crit	Wistar	1	1	1
	msP	6	1	2

Based on the sum of normalized scores (*z*-scores) assigned to each criterion (Figure 4B), we obtained a GAS for individual rats. The average of this addiction score for each subpopulation was negative for the 0 and 1 crit groups (0crit = −2.07 and 1crit = −0.08) and positive for 2 and 3 criteria groups (2crit = 1.73 and 3crit = 3.1; Figure 4C). We found a main effect for addiction scores among the criteria subgroups as revealed by one-way ANOVA (*F*_(3,59)_ = 37.81; *p* < 0.001). Neuman-Keuls *post hoc* test showed a significant difference between each addiction score (0crit vs. 1crit, 2crit and 3crit *p* < 0.001; 1crit vs. 2crit and 3crit *P* < 0.001; 2crit vs. 3crit *p* < 0.05).

### Differences in the Three Measures of AUD-Like Behavior in 0crit, 1crit, 2crit and 3crit Groups

One-way ANOVA applied to persistence in alcohol seeking revealed a significant difference between groups (*F*_(3,59)_ = 14.83; *p* < 0.001; Figure 4D). The slope of active lever pressing during the no-drug-period was about nil in 0crit (−0.05 ± 0.33) and progressively increased as a function of the criteria met: 1crit (0.85 ± 0.38), 2crit (2.76 ± 0.46) and 3crit (3.87 ± 0.65). Neuman-Keuls *post hoc* test showed that 3crit rat exhibited higher active lever pressing slope compared to 0crit (*p* < 0.001) and 1crit (*p* < 0.001) groups but not compared to 2crit group (*p* = ns). The 1crit rats differed as well from 2crit rats (*p* < 0.001) but not from 0crit group (*p* = ns).

In the motivation for alcohol (Figure 4E), one-way ANOVA revealed a significant between groups difference in the breakpoint (*F*_(3,59)_ = 11.71; *p* < 0.001). As shown by Neuman-Keuls *post hoc* test, 3crit rats exhibited higher breakpoint (43.5 ± 1.4) compared to the 0crit (26.48 ± 1.9; *p* < 0.001) and 1crit (33.36 ± 2.18; *p* < 0.01) groups but not compared to 2crit rats (44 ± 3.27; *p* = ns). In addition, the 2crit rats differed from 0crit (*p* < 0.01) and 1crit (*p* < 0.05) rats but not from 3crit rats (*p* = ns).

Kruskal Wallis H test of punished alcohol seeking revealed an overall effect of groups (H 3, *N* = 63 = 25,064 *p* < 0.001; Figure 4F). Multiple comparisons showed that 3crit rats presented a higher rate of punished reinforcers (71.32 ± 5.27) compared to 0crit (18.92 ± 3.4; *p* < 0.001), 1crit (47.01 ± 6.4; *p* < 0.01) and 2crit (46.86 ± 6.75; *p* < 0.05) rats. Moreover, 2crit group differed from 0crit group (*p* < 0.01) and 1crit group differed from 0crit group (*p* < 0.01).

### Alcohol Consumption and AUD-Like Behavior

**Figure 5:**
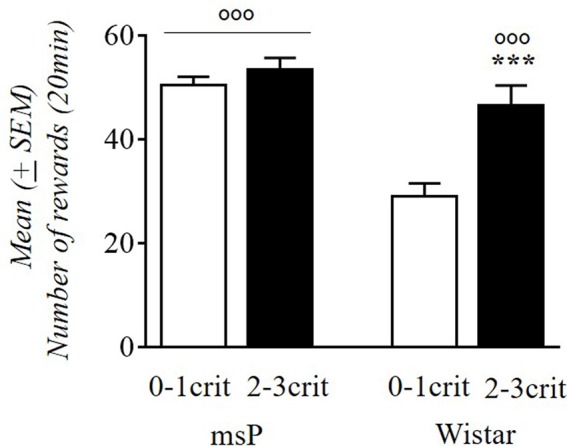
Alcohol rewards (average days 42–44) comparing 0–1crit vs. 2–3crit in msP and Wistar rats. Values are presented as mean (±SEM) ^***^p < 0.001 significant as compared to 0–1crit and 2–3crit Wistar rats. °°°*p* < 0.001 significant as compared to 0–1crit Wistar rats and 0–1crit, 2–3crit msP rats.

We evaluated the relationship between alcohol intake in msP and Wistar rats with the propensity to develop addiction-like behavior vs. resistance (2–3crit vs. 0–1crit) (Figure 5). Factorial ANOVA revealed a significant effect of genotype (*F*_(1,59)_ = 20.77; *p* < 0.001) and criteria subgroup (*F*_(1,59)_ = 11.00; *p* < 0.001) and a significant interaction between genotype and the criteria subgroup on alcohol intake (*F*_(1,59)_ = 5.53; *p* < 0.05). Newman-Keuls *post hoc* test showed that 0–1crit Wistar rats had lower levels of alcohol intake compared to 2–3crit Wistar rats (*p* < 0.01) while the 0–1crit msP rats did not differ in alcohol intake from the 2–3crit msP rats (*p* = ns). Moreover, 0–1crit Wistar rats differ from 0 to 1crit msP (*p* < 0.001) while there is not a statistically significant difference between 2–3crit Wistar and 2–3crit msP rats on alcohol intake (*p* = ns).

### Anxiety and the Vulnerability to Develop Addiction-Like Behavior

In the literature, high anxiety behavior has been associated with the development of drug addiction (Stewart and Conrod, 2008; Ipser et al., 2015). As revealed by Pearson’s analysis, a significant (*r* = −0.38, *p* < 0.05) negative correlation between alcohol intake during ethanol self-administration and the percentage of time spent in the open arms was found in msP rats in which high levels of alcohol drinking were associated with higher anxiety. No significant correlation was detected in Wistars (*r* = −0.04, *p* = ns). We also compared the three addiction-like criteria with the percentage of time spent in open arms of the EPM. Pearson’s analysis indicated no correlation between anxiety levels and the three addiction-like criteria in msP (persistence in alcohol seeking: *r* = 0.084; *p* = ns, motivation for alcohol: *r* = −0.137; *p* = ns, resistance to punishment: *r* = −0.052; *p* = ns) or Wistar rats (persistence in alcohol seeking: *r* = 0.072; *p* = ns, motivation for alcohol: *r* = 0.066; *p* = ns, resistance to punishment: *r* = −0.29; *p* < 0.001).

## Discussion

Modeling human AUDs in rodents has been a challenge in preclinical studies since alcohol, unlike cocaine or opioids, is a weak reinforcer and requires protracted exposure for the development of dependence (Spanagel and Hölter, 1999). A valid animal model that attempts to reflect the human condition in addiction should capture multiple aspects that characterize substance use disorders (SUDs) such as compulsive drug seeking and taking, increased motivation for the drug, and continued intake despite negative consequences (Hopf and Lesscher, 2014).

In this study, based on the DSM-IV/5 diagnostic criteria, we used a multidimensional model including different behavioral features of addiction to characterize an alcohol addiction prone phenotype in rats. We also sought to evaluate the role of genetic predisposition to excessive drinking in shaping individual variability in developing alcohol addictive like-behavior by comparing alcohol preferring msP rats with non-preferring progenitor Wistar rats.

Our data showed an enhanced propensity of the msP rats to exhibit higher scores in the three defined criteria for individual vulnerability to AUD, as measured in: (1) persistence in alcohol seeking when alcohol is not available; (2) motivation for alcohol in a PR schedule of reinforcement; and (3) resistance to punishment when alcohol delivery is anticipated by a foot-shock. The percentage of rats positive in all three criteria was approximately 13% with msP rats representing the majority of the 3crit group compared to Wistar rats, 9.52% vs. 3.17%, respectively. The percentage of rats characterized as 3crit is similar to the small proportion of individuals that develop alcohol dependence after protracted exposure (Anthony et al., 1994; Wagner and Anthony, 2002). The 2crit group was also enriched in msP rats, while Wistars constituted the majority of the 0crit and 1crit groups. The inter-individual variability we observed was present not only between but also within genotypes demonstrating that this protocol is a suitable approach to study individual differences in alcohol dependence in largely homogeneous rat populations.

Persistence of alcohol seeking was evaluated daily throughout the training period. We ran a cluster analysis to identify a subgroup of rats that increased their response during the drug free period over time, as other laboratories have failed to capture this behavioral criterion (Waters et al., 2014). By clustering rats based on their persistence in lever pressing in the absence of alcohol availability, we were able to determine a subpopulation of rats (38% of msP and 26% of Wistars) that were highly persistent in lever pressing. Recent studies on rodent models of AUDs have interpreted the intrasession drug free period as a measure of alcohol seeking behavior (Jadhav et al., 2017; Radke et al., 2017). However, in those studies persistence to response when alcohol was not available was assessed only in three to five sessions making it difficult to evaluate how uncontrolled drug seeking develops over time (Belin et al., 2016). Here, in line with earlier studies using the 0/3crit model in cocaine use, we demonstrated that persistence of alcohol seeking is a trait that develops over time but only in a subset of animals.

In assessing motivation for alcohol by using the PR schedule of reinforcement, we found that msP rats, compared to Wistar rats, exhibited increased motivation to self-administer alcohol as shown by higher breakpoints during the PR session (Ciccocioppo et al., 2006). This schedule, more than the persistence of alcohol seeking or resistance to punishment, is linked to consummatory behavior, a trait used to select msP rats. It is not surprising, therefore, to observe such a remarkable difference from non-selected Wistars. Previous studies have also demonstrated that the breakpoint is sensitive to genetic selection procedures (Czachowski and Samson, 2002).

As described in the DSM-IV and DSM-5, compulsive drug seeking or drug-taking despite negative consequences is another hallmark of drug dependence (American Psychiatric Association, 2000, 2013).

A recent preclinical model developed in Cambridge laboratories, has been able to identify a subset of vulnerable individuals that display compulsive alcohol seeking in the face of punishment (Giuliano et al., 2018). By using probabilistic footshock punishment of the seeking response in a seeking-taking chained schedule of reinforcement they were able to distinguish the punisher from the reward. Indeed, it has been demonstrated that when the footshock co-occurs with the drug delivery its effectiveness as a punisher is reduced (Dickinson and Pearce, 1976; Pelloux et al., 2007). Several models use footshock punishment (various shock intensities have been used) paired with the delivery of a constant dose of alcohol (Seif et al., 2013; Jadhav et al., 2017; Radke et al., 2017; Augier et al., 2018). To distinguish the punishing from the reinforcing proprieties of alcohol here, rats were punished with 0.3 mA footshock that preceded alcohol taking response without pairing the shock with ethanol delivery. Results showed that punishing operant responding markedly decreased alcohol seeking in both msP and Wistar rats. This phenomenon was previously described when footshock punishment was used in rats trained in cocaine self-administration (Deroche-Gamonet et al., 2004; Kasanetz et al., 2010). However, individual animals showed different behavioral suppression levels that spanned from 0 to 100% of baseline. Most importantly, in both lines a subgroup of rats continued to self-administer alcohol despite the negative consequences of footshock.

Consistent, with previous works adopting the 0–3crit model of cocaine (Belin et al., 2008, 2011; Kasanetz et al., 2013; Cannella et al., 2017, 2018) and alcohol (Radke et al., 2017) addiction, here we used a single foot-shock session to assess resistance to punishment. As demonstrated by other studies, using multiple punishment sessions could have been an alternative to better separate the population in shock-resistant and shock-sensitive subgroups (Seif et al., 2013; Augier et al., 2018; Giuliano et al., 2018; Marchant et al., 2018). However, a single shock-test session is also informative of individual resistance to punishment. Moreover, a recent work demonstrated that in the context of the 0–3crit model applied to alcohol, multiple shock tests can compress rather than increase the range of distribution of resistance to punishment. This study also revealed that resistance to punishment of the 0crit to 3crit groups decreases over time, although the groups differences are maintained (Jadhav et al., 2017).

As a second level analysis, by summing the normalized score (*z*-score) applied to single criteria the GAS for individual subject was calculated. Results showed that each criteria group differed significantly in GAS. Specifically, GAS was negative in the 0crit/1crit groups identified as resistant whereas it was positive in the 2crit/3crit groups that were characterized as prone to develop an alcohol dependent-like phenotype. We found a high correlation between the GAS and the distribution of the animals within the three criteria, indicating the interdependence between these two measures.

Epidemiologic studies have suggested that genes play an important role in the vulnerability to alcohol abuse and the subsequent risk to develop alcohol dependence (Crabbe et al., 2006; Mayfield et al., 2008; Schuckit, 2009). In recent years there has been considerable debate on whether genetically-predisposed alcohol drinking rodents may adequately model AUDs. To this end an ideal genetic animal model of alcoholism should carry the same genetic traits linked to alcoholism in humans, and ideally those traits should correlate with the expression of similar subphenotypic characteristics. One of the genetic traits of msP rats is the over-expression of the corticotropin-releasing factor system, linked to the presence of two single nucleotide polymorphisms (SNPs) of the CRF1 receptor (CRF1-R) gene leading to receptor overexpression (Hansson et al., 2006; Ayanwuyi et al., 2013; Cippitelli et al., 2015). Polymorphisms at level of the promoter region of the CRF1-R gene have been reported in humans with AUDs, suggesting a genetic trait in common with msP rats (Treutlein et al., 2006). Notably, resembling a large subset of alcoholic patients, msP rats also drink excessive amounts of alcohol (7–8 g/kg/day) and exhibit an anxious and depressive-like behavioral phenotype. In msP rats this phenotype is, at least in part, linked to a hyperactive CRF1-R function (Schuckit and Hesselbrock, 1994; Grant et al., 2004; Ayanwuyi et al., 2013; Cippitelli et al., 2015). Here, we confirmed that, compared to unselected Wistars, the msP rats have a higher basal level of anxiety. However, when we attempted to associate open arm time (a measure of anxiety) with animal distribution within the 3 criteria, no significant correlations were detected. This, together with earlier findings, suggest that high anxiety is linked to a genetic predisposition to excessive drinking but not with propensity to develop alcohol abuse traits (compulsive-like alcohol use, persistence in seeking, and motivation). Dilleen et al. (2012) demonstrated recently that a high anxiety trait predicts loss of control over cocaine, but not heroin self-administration, suggesting that outcome may depend on the psychoactive drug used.

msP rats represented the majority of subjects belonging to the 2crit and 3crit groups. However, Wistar rats satisfying the 2/3crit, self-administered as much alcohol as the msPs rats, and significantly more than 0/1crit Wistars. The msP rats in the 0/1crit group consumed the same high amount of alcohol of the 2/3crit group. These data indicate that a genetic predisposition to ethanol preference and excessive drinking may not be necessarily associated with a propensity to develop addictive-like traits modeled by the 0/3crit paradigm used here. Interestingly, in humans it has been shown that a large number (about 90%) of people with excessive drinking habits do not meet the criteria for alcohol dependence (Esser et al., 2014). Our results are therefore in line with human data and with results from another recent study which found that the propensity for P alcohol preferring rats to drink high levels of alcohol was dissociable from the development of compulsive alcohol seeking (Giuliano et al., 2018). Together these data suggest that the study of individual vulnerability is an important approach to investigate AUD, as it is able to dissociate excessive alcohol drinking from the propensity to develop the dependence, thus mimicking the human condition. Ultimately, the study of individual vulnerability and the employment of this model in pharmacological studies may help refine the investigation of novel chemical entities for AUD by exploring their efficacy on specific traits not limited to drinking, and that more closely mimic the human condition.

## Author Contributions

AD and RC were responsible for the study concept and design. AD performed the experiments. AD, NC, RC, ED and SS assisted with the data analysis, interpretation of findings and drafted the manuscript. All authors critically reviewed the content and approved the final version for publication.

## Conflict of Interest Statement

The authors declare that the research was conducted in the absence of any commercial or financial relationships that could be construed as a potential conflict of interest.
